# Light and Heat

**Published:** 1883-05

**Authors:** 


### Popular Science Department.

### LIGHT AND HEAT.

The amount of light given out by a gas flame depends upon the temperature to which the particles of solid carbon in the flame are raised, and Dr. Tyndall has shown that of the radiant energy set up in such a flame, only the one-twenty-fifth part is luminous; the hot products of combustion carry off at least four times as much energy as is radiated, so that not more than one-hundredth part of the heat evolved in combustion is converted into light.—*Sci. Amer.*

